# Application of single-cell multi-omics approaches in horticulture research

**DOI:** 10.1186/s43897-023-00067-y

**Published:** 2023-09-26

**Authors:** Jun Zhang, Mayra Ahmad, Hongbo Gao

**Affiliations:** Joint Center for Single Cell Biology, School of Agriculture and Biology, Shanghai Jiao Tong University, Shanghai, 200240 China

**Keywords:** Single-cell, Multi-omics, High throughput sequencing, Cell atlas, Developmental trajectory

## Abstract

Cell heterogeneity shapes the morphology and function of various tissues and organs in multicellular organisms. Elucidation of the differences among cells and the mechanism of intercellular regulation is essential for an in-depth understanding of the developmental process. In recent years, the rapid development of high-throughput single-cell transcriptome sequencing technologies has influenced the study of plant developmental biology. Additionally, the accuracy and sensitivity of tools used to study the epigenome and metabolome have significantly increased, thus enabling multi-omics analysis at single-cell resolution. Here, we summarize the currently available single-cell multi-omics approaches and their recent applications in plant research, review the single-cell based studies in fruit, vegetable, and ornamental crops, and discuss the potential of such approaches in future horticulture research.

**Graphical Abstract:**

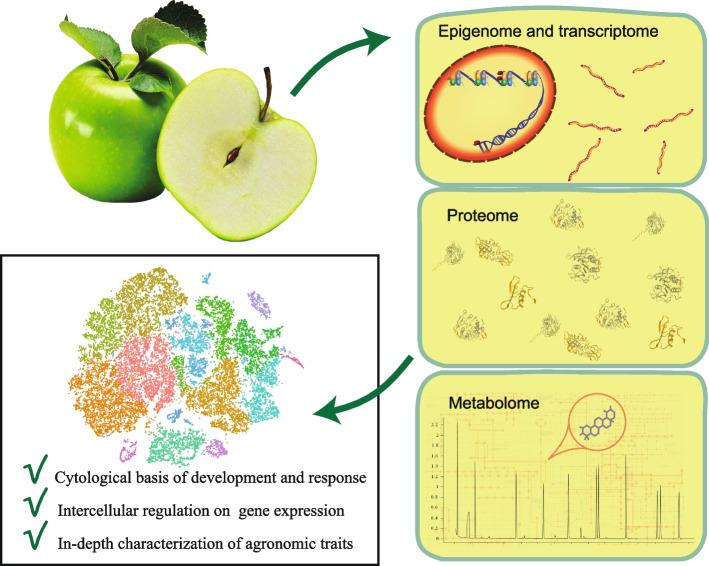

## Introduction

Cell heterogeneity is the driving force of morphological diversity and functional differentiation in multicellular organisms. Cell-specific regulation mechanisms control plant cell differentiation, metabolic partitioning, and environmental response, processes that need to be characterized at single-cell resolution (Ryu et al. 2021; Seyfferth et al. 2021). Recent technological advances have enabled multi-omics, including transcriptomic, epigenomic, and metabolomic, analyses of isolated single plant cells, which have been used to study many aspects of plants and are emerging as powerful tools for dissecting the precise and sophisticated intercellular regulatory mechanisms. Horticulture plants include many plant families and species, and exhibit agronomic trait-related developmental diversity at the whole-plant, organ, tissue, and cellular levels. Therefore, the emerging single-cell approaches are particularly suitable for clarifying the development of horticulturally important plants and for uncovering the novel intercellular regulatory mechanisms employed by these plants.

In this review, the single-cell multi-omics approaches currently available for plant research are summarized, and the application areas of plant single-cell multi-omics studies in recent years are discussed. More importantly, we systematically summarized the studies that applied single-cell multi-omics methods to horticulture plants. Based on the current technical developments and their applications in plant research, several horticulture related questions are proposed that can be addressed by single-cell multi-omics approaches in the future.

## Advances in single-cell multi-omics approaches

**Fig. 1:**
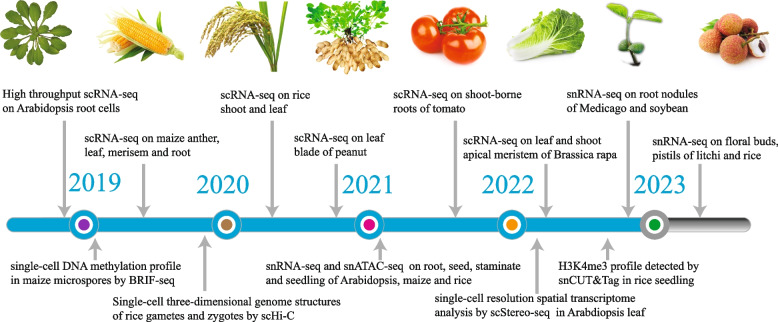
Timeline of pioneer research on plant single-cell transcriptomics and epigenomics

Omics studies provide global information on gene regulation, protein function, and metabolic composition. In recent years, multi-omics techniques have evolved rapidly, enabling genome-wide profiling at the single-cell resolution. In this section, we summarize the single-cell multi-omics tools that have been successfully applied in plant research. The plant species and tissues/organs investigated using each approach are listed in Table 1, and pioneer studies in several plant species are highlighted in Fig. 1.

**Table 1:** Single-cell multi-omic approaches in plant research

	**Methods**	**Species**	**Organ/tissue**	**reference**
**Transcriptomics**	Drop-seq	*Arabidopsis thaliana, Populus alba* × *Populus glandulosa, Catharanthus roseus*	seedling, root, xylem, leaf	(Shulse et al. 2019; Turco et al. 2019; Li et al. 2021; Li et al. 2023)
	10 × Genomics	*Arabidopsis thaliana*, *Arachis hypogaea, Brassica rapa*, *Camellia sinensis, Fragaria vesca*, *Medicago truncatula*, *Nicotiana attenuate, Oryza sativa, Populus alba, Zea mays, Marchantia polymorpha, Bombax ceiba, Gossypium hirsutum, Catharanthus roseus, Phyllostachys edulis, Hevea brasiliensis, Populus trichocarpa, Liriodendron chinense*	root, cotyledon, leaf, ear inflorescence, shoot, stem, root nodule, corolla limbs and throat cups, ovule, gemmae and thalli, inner ovary wall, stem-differentiating xylem	(Bai et al. 2022; Chen et al. 2021; Denyer et al. 2019; Gala et al. 2021; Graeff et al. 2021; Guo et al. 2022; Hou et al. 2021; Jean-Baptiste et al. 2019; Kang et al. 2022; Liu et al. 2021a; Liu et al. 2021b; Liu et al. 2020; Lopez-Anido et al. 2021; Ortiz-Ramírez et al. 2021; Otero et al. 2022; Ryu et al. 2019; Shahan et al. 2022; Sun et al. 2022; Wang et al. 2022; Wang et al. 2021; Wendrich et al. 2020; Xu et al. 2021; Ye et al. 2022; Zhang et al. 2021; Zhang et al. 2019; Kim et al. 2021; Cao et al. 2023; Wang et al. 2023; Zhu et al. 2023b; Liu et al. 2022; Ding et al. 2023; Qin et al. 2022; Li et al. 2023; Sun et al. 2023; Cheng et al. 2023; Liang et al. 2023; Tung et al. 2023)
	Microwell based scRNA-seq	*Arabidopsis thaliana, Oryza sativa*	callus, inflorescence	(Zhai and Xu 2021; Zong et al. 2022)
	CEL-seq2	*Zea mays*	anther, shoot apical meristems	(Nelms and Walbot 2019; Satterlee et al. 2020)
	mcSCRB-seq	*Solanum lycopersicum*	shoot-borne root	(Omary et al.^ 2022^)
	SMART-seq	*Arabidopsis thaliana*	root meristem, lateral root primordium	(Roszak et al. 2021; Serrano-Ron et al. 2021)
	snRNA-seq	*Arabidopsis thaliana, Medicago truncatula, Oryza sativa, Zea mays, Litchi chinensis, Glycine max *	root, endosperm, leaf, floral meristems, seedlings, pistil, bud, root nodules	(Cervantes-Pérez et al. 2022; Farmer et al. 2021; Li et al. 2023a; Marand et al. 2021; Neumann et al. 2022; Picard et al. 2021; Wang et al. 2023; Yang et al. 2023; Liu et al. 2023)
	1cell-DGE	*Physcomitrella patens*	leaf	(Kubo et al. 2019)
	scStereo-seq	*Arabidopsis thaliana*	leaf	(Xia et al. 2022)
	Quartz-Seq2	*Arabidopsis thaliana*	callus	(Ogura et al.^ 2023^)
	PHYTOMap	*Arabidopsis thaliana*	root	(Nobori et al. 2023)
	MARS-seq2.0	*Eucalyptus grandis, Trochodendron aralioides*	stem-differentiating xylem	(Tung et al. 2023)
**Epigenomics**	BRIF-seq	*Zea mays*	microspore	(Li et al. 2019b)
	scATAC-seq	*Arabidopsis thaliana, Oryza sativa, Zea mays*	root, seedling, axillary buds, embryonic root, staminate, pistillate inflorescence, crown root	(Dorrity et al. 2021; Farmer et al. 2021; Feng et al. 2022; Marand et al. 2021)
	sci-ATAC-seq	*Arabidopsis thaliana*	root	(Tu et al. 2022)
	snCUT&Tag	*Oryza sativa*	seedling	(Ouyang et al. 2022)
	scHi-C	*Oryza sativa*	sperm, egg, zygote	(Zhou et al. 2019)
**Metabolomics**	Nano-LC–ESI–MS	*Torenia hybrida*	petal cell	(Kajiyama et al. 2006)
	Nano-ESI–MS	*Catharanthus roseus, Pelargonium zon*, *Vicia faba*	single leaf, stem, petal cell, parenchyma, epidermal, idioblast, and laticifer cells	(Lorenzo Tejedor et al. 2012; Shimizu et al. 2015; Tejedor et al. 2009; Yamamoto et al. 2019, 2016)
	LAESI-MS	*Allium cepa, Narcissus pseudonarcissus*	single epidermal cell of bulb	(Shrestha and Vertes 2009)
	UV-MALDI-ToF–MS	*Tulipa suaveolens*	leaf and bulb	(Gholipour et al. 2012)
	LDI-ToF–MS	*Arabidopsis thaliana, Hypericum perforatum, Hypericum reflexum *	individual dark glands from petals, leaves, glandular trichomes	(Hölscher et al. 2009)
	CE-MS	*Allium cepa*	epidermal cell	(Huang et al. 2021)
	UPLC-MS	*Catharanthus roseus*	leaf	(Li et al. 2023)

### Transcriptomics

Transcriptomics at the single-cell level can enhance cell-type resolution and reveal gene regulatory networks. Initially, researchers isolated single cells from *Arabidopsis thaliana* roots and used single-cell transcriptomic to analyze the formation of root stem cells during regeneration (Efroni et al. 2016). Subsequently, advances in single-cell isolation techniques, microfluidics, next-generation sequencing (NGS), and bioinformatics led to the development of high-throughput single-cell transcriptomic methods (Jaitin et al. 2014; Klein et al. 2015; Kolodziejczyk et al. 2015; Macosko et al. 2015; Zheng et al. 2017). To date, droplet-based single-cell RNA sequencing (scRNA-seq) methods, such as inDrop and Drop-seq (Macosko et al. 2015), and the Chromium 10X platform have been most widely adopted to study the plant single-cell transcriptome landscape, for example, in *Arabidopsis* roots (Denyer et al. 2019; Dorrity et al. 2021; Farmer et al. 2021; Jean-Baptiste et al. 2019; Ryu et al. 2019; Shahan et al. 2022; Zhang et al. 2019), cotyledons (Liu et al. 2020), and leaves (Kim et al. 2021; Liu et al. 2022), as well as rice (*Oryza sativa*) leaves and roots (Liu et al. 2021b; Wang et al. 2021), because these approaches are high-throughput and facilitate low-cost cell processing. Microwell based scRNA-seq methods have also been used in plants (Zhai and Xu 2021; Zong et al. 2022). A single plant cell can be isolated manually or by fluorescence-associated cell sorting (FACS) into each well of a plate, and scRNA-seq libraries can be generated by Cell Expression by Linear amplification and sequencing (CEL-seq2) (Hashimshony et al. 2016), molecular crowding single-cell RNA barcoding and sequencing (mcSCRB-seq) (Bagnoli et al. 2018), Quartz-Seq2 (Sasagawa et al. 2018), MARS-seq2.0 (Keren-Shaul et al. 2019), and SMART-seq related methods, including SMART-seq2 (Picelli et al. 2014), SMART-seq3 (Hagemann-Jensen et al. 2020), SMART-seq3xpress (Hagemann-Jensen et al. 2022), and FLASH-seq (Hahaut et al. 2022), which have been successfully used in plant studies with high sensitivity and flexibility (Nelms and Walbot 2019; Ogura et al. 2023; Omary et al. 2022; Roszak et al. 2021; Tung et al. 2023).

Because of the presence of cell wall, single plant cells are difficult to isolate and manipulate, which impedes the application of scRNA-seq in mature and lignified plant organs. Single-nucleus RNA-seq (snRNA-seq), which was originally developed for difficult-to-dissociate samples or frozen specimens in animals (Kalish et al. 2020; Liang et al. 2019), has also been applied to plants, for example, to study the endosperm and leaf of *Arabidopsis* (Picard et al. 2021; Wang et al. 2023). Comparative analyses have shown that snRNA-seq provides an alternative and effective means to identify different cell types, demonstrating conserved representation of the transcriptome in the cytoplasm and nucleus (Bakken et al. 2018; Ding et al. 2020; Farmer et al. 2021; Kulkarni et al. 2019; Mereu et al. 2020). Apart from snRNA-seq, spatial transcriptomics, which captures mRNAs from spatially distinct tissue sections, can also overcome the difficulty of single-cell isolation, along with an additional benefit of preserving the three-dimensional (3D) location information. Previously, Kubo et al. established single cell-digital gene expression (1cell-DGE), a method that uses micromanipulation, to extract the contents of individual living cells in intact tissue while recording their positional information. With 1cell-DGE, the authors detected differentially expressed genes (DEGs) during the reprogramming of leaf cells of the moss *Physcomitrella patens* (Kubo et al. 2019). Subsequently, using DNA nanospheres, single-cell Spatial Enhanced REsolution Omics sequencing (scStereo-seq) was developed, enabling the determination of spatially-resolved transcriptome at the single-cell level. The examination of *Arabidopsis* leaves by scStereo-seq could clearly define cell boundaries, classify specific cell types, and describe the developmental trajectory of vascular and guard cells (Xia et al. 2022). Plant HYbridization-based Targeted Observation of gene expression map (PHYTOMap) enables the spatial analysis of gene expression at the single-cell level in whole-mount plant tissue in a transgene-free manner. The major cell types in *Arabidopsis* roots have successfully been identified by applying PHYTOMap to simultaneously analyze 28 cell-type-specific marker genes (Nobori et al. 2023).

### Epigenomics

Epigenetic factors, including DNA methylation, chromatin accessibility, histone modifications, and 3D genome structure, can regulate gene activity and play important roles in developmental programing during cell differentiation (Wen and Tang 2022). Genome-wide profiling of DNA methylation is usually performed using bisulfite conversion related methods. Although such methods cause DNA damage and result in the loss of genomic information, several methods, including single-cell bisulfite sequencing (scBS-seq) (Smallwood et al. 2014), single-nucleus methylcytosine sequencing (snmC-seq) (Luo et al. 2018), and single-cell combinatorial indexed assay for the assessment of DNA methylation (sciMET) (Mulqueen et al. 2018), have been set up and managed to perform DNA methylation assessment at single-cell resolution in mammals. Similar methods can potentially be adapted for plants to study DNA methylation at the single-cell level. For example, bisulfite-converted randomly integrated fragments sequencing (BRIF-seq) was developed and used to study the single microspores of maize (*Zea mays*), which produced high read-mapping rates and genome coverage, enabling the identification of heterogeneous sites (Li et al. 2019).

Chromatin accessibility at the single-cell level can be detected by single-cell DNase I hypersensitive site sequencing (scDNase-seq), single-cell combinatorial indexing assay for transposase-accessible chromatin with sequencing (sci-ATAC-seq), and single-cell assay for transposase-accessible chromatin with high-throughput sequencing (scATAC-seq) (Buenrostro et al. 2015; Cusanovich et al. 2015; Jin et al. 2015). The scATAC-seq data can be integrated with scRNA-seq data to reveal novel gene regulation mechanisms and to precisely identify rare cell types. For example, combining scATAC-seq with snRNA-seq enabled the construction and analysis of a cis-regulatory element map from multiple maize organs, thereby revealing cell-type-specific cis-regulatory elements that are closely associated with phenotypic variation (Marand et al. 2021). Similar approaches revealed the dynamic changes in chromatin accessibility during cell differentiation and development in *Arabidopsis* root, and determined the transcriptomes of novel root cell types (Dorrity et al. 2021; Farmer et al. 2021). Recently, an alternative low-cost and high-throughput approach, sci-ATAC-seq, was developed for single-cell epigenome profiling in *Arabidopsis* root, which identified 24 cell clusters with unique transcription, chromatin, and cis-regulatory signatures (Tu et al. 2022).

Cleavage Under Targets and Tagmentation (CUT&Tag) technology has been used widely to profile genome-wide histone modifications and shows high sensitivity on limited samples. Recently, single-nucleus CUT&Tag (snCUT&Tag) has been reported for determining the H3K4me3 profile in rice seedlings (Ouyang et al. 2022), indicating that snCUT&Tag can be applied to dissect epigenetic heterogeneity in single plant cells.

DNA/protein modifications, nucleosome positioning, and protein-DNA interactions act together to shape the overall 3D genome structure. Single-cell high-throughput chromatin capture (scHi-C) techniques can be used to study the 3D genome structure of single cells (Nagano et al. 2013; Stevens et al. 2017). The use of scHi-C provided a spatial chromatin basis for zygotic genome activation (ZGA) and epigenetic regulation in plants (Zhou et al. 2019). Updated approaches with higher throughput and sensitivity, such as single-cell combinational index Hi-C (sciHi-C) (Ramani et al. 2017) and Dip-C (Tan et al. 2018), have been developed in mammals, which have enormous potential for related studies in plants.

### Proteomics

Proteins are the primary executor of biological functions. Thus, high resolution dissection of proteome and post-translational modifications (PTM) at the single-cell level can reveal the exact molecular mechanisms of intercellular regulations. In a pioneer study that combined microcapillary-based single-cell sampling with matrix-assisted laser desorption/ionization time-of-flight mass spectrometry (MALDI-TOF MS) and nanoscale liquid chromatography-tandem mass spectrometry (nanoLC-MS/MS), several proteins were identified from a single *Arabidopsis* S-cell (a glucosinolate-rich cell type in the flower stalk), demonstrating that proteomic analysis can be applied at the single-cell level to identify the most abundant proteins or biomarkers and to reveal cell-type-specific differences (Koroleva and Cramer 2011). Recent improvements in MS-based methods have significantly increased their sensitivity and mass accuracy and have enabled the measurement of several thousand proteins in small subpopulations of cells and even in single mammalian cells (Bennett et al. 2023). In *Arabidopsis*, a high quality quantitative atlas of the transcriptomes, proteomes, and phosphoproteomes of different tissues and cell types was also constructed recently (Mergner et al. 2020). Thus, proteomic analysis at the single-cell resolution is foreseeable in both animal and plant research (Yan et al. 2022).

### Metabolomics

Single-cell metabolomics is of paramount interest, given that the sum of the functions and interactions of individual cells translates into the function of tissues, organs, and whole organism. At present, research on single-cell MS methods focuses on the development of ionization techniques and the corresponding sample pre-treatment methods. There are several types of single-cell MS methods with varying ionization techniques: MALDI imaging (Kaspar et al. 2011), nano-electrospray ionization mass spectrometry (nanoESI-MS), laser desorption ionization mass spectrometry (LDI-MS) (Hölscher et al. 2009), and secondary ion mass spectrometry (SIMS) (Moore et al. 2012). MALDI imaging is the most extensively used MS Imaging (MSI) technique (Bjarnholt et al. 2014; Hansen and Lee 2018), and has been used for the molecular imaging of plant tissues (Kaspar et al. 2011). Besides MALDI, SIMS and ambient ionization techniques (Venter et al. 2008), such as desorption ESI (DESI) (Takáts et al. 2004) and laser ablation ESI (LAESI) (Nemes et al. 2009), are the other popular sampling probes that have been employed in MS-based imaging measurements. Furthermore, integrating novel microsampling techniques with MALDI-MS and ESI–MS analyses can facilitate the development of single-cell metabolomics in plants. For example, anthocyanins in single petal cells of wishbone flower (*Torenia hybrida*) were analyzed using laser microsampling (Kajiyama et al. 2006). Additionally, in onion (*Allium cepa*) and daffodil (*Narcissus pseudonarcissus*), LAESI-MS analysis of individual cells in the bulb epidermis led to the identification of 35 metabolites (Shrestha and Vertes 2009). Recently, the combined application of a cell pressure probe and ultraviolet (UV)-MALDI-TOF MS allowed in situ picoliter-scale sampling and metabolite profiling in the single leaf and bulb cells of tulip (*Tulipa suaveolens*) (Gholipour et al. 2012). Furthermore, direct single-cell analysis via nano-ESI tip aspiration under a video-microscope has been developed to measure metabolites in their native environment. Aspiration of Wilde Malva (*Pelargonium zonale*) leaf stalk single-mesophyll-cell contents using a nano-ESI tip, followed by MS, revealed over 1,000 features from a sample of 1–5 pL in volume (Tejedor et al. 2009). Similarly, a nano-ESI tip was used to extract the cellular contents from live single cells in the leaf, stem, and petal tissues of Wilde Malva. Subsequently, nanoESI-MS enabled the identification of terpenoid hydrocarbons and isoprenoids, among several other compounds, reported for the first time in Wilde Malva (Lorenzo Tejedor et al. 2012). In addition, a combination of laser microdissection (LMD) sampling and LDI-ToF–MS allowed the localization of specialized metabolites at a resolution of 10 μm in St. John’s wort (*Hypericum perforatum*), *Hypericum reflexum*, and *Arabidopsis* (Hölscher et al. 2009). Recently, with the advances in sample separation and MS techniques, single-cell capillary electrophoresis mass spectrometry (CE-MS) has become a promising platform for analyzing cellular contents and probe cell heterogeneity (DeLaney et al. 2018; Kristoff et al. 2020; Yan et al. 2022). Hundreds of metabolites in a single red-onion cell have been successfully separated and putatively identified using an online single-cell CE-MS platform (Huang et al. 2021). Although progress has been made in the development of single-cell MS approaches, none of these methods uses chromatographic separation before MS analysis, greatly limiting the accurate structural assignment and quantification of metabolites. To address these limitations, ultra-high liquid chromatography-mass spectrometry (UPLC-MS) was used to perform the single-cell metabolomics analysis of individual leaf cells of *Catharanthus roseus*, which led to the identification of several metabolites, including anhydrovinblastine (AHVB), vinblastine, catharanthine, serpentine, vindoline, and secoiridoid secologanin (Li et al. 2023b).

### Multi-omics

Single-cell multi-omics technologies typically measure multiple types of molecules obtained from the same individual cell. The development and implementation of single-cell technologies for multi-omics measurements of genome, transcriptome, epigenome, proteome, and metabolome are opening tremendous opportunities for enhancing our mechanistic understanding of cellular phenotypes and higher-order biological systems. To date, a number of assays for single-cell multi-omics profiling have been developed to profile DNA sequences, gene expression, and epigenetic information simultaneously at the single-cell level in mammalian systems (Bai et al. 2021), but the application of single-cell multi-omics in plants is limited. Currently, the integration of snRNA-seq and scATAC-seq allows the association of chromatin accessibility with gene expression at the single-cell level in *Arabidopsis* and maize organs (Dorrity et al. 2021; Farmer et al. 2021; Marand et al. 2021). The combined application of scRNA-seq and single-cell MS revealed the monoterpene indole alkaloid (MIA) biosynthetic pathway in *C. roseus* (Li et al. 2023b). With the advent of single-cell multi-omics technologies, it has become increasingly possible to perform simultaneously study the transcriptome, epigenome, proteome, and/or metabolome profiles of the same single cell in plants (Chappell et al. 2018; Efremova and Teichmann 2020; Ma et al. 2020; Song et al. 2019), which can provide sufficient information for exploring and identifying cell characteristics.

## Application areas of single-cell multi-omics approaches in plant research

**Fig. 2:**
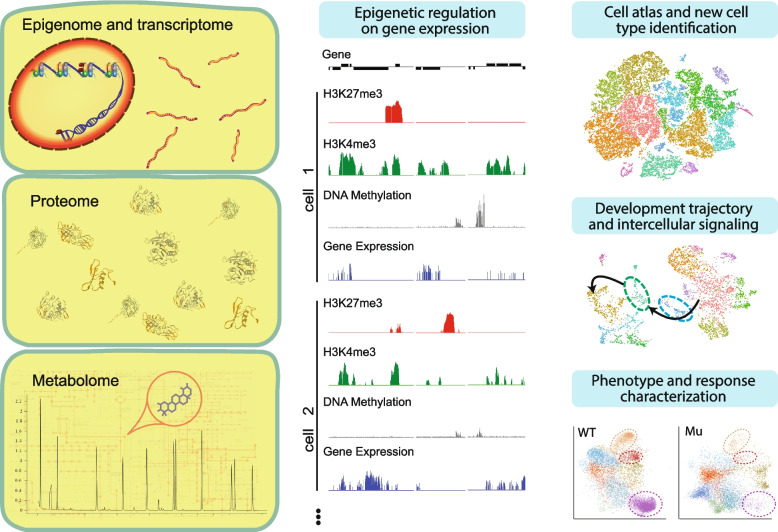
Application areas of single-cell multi-omics approaches in plant research

Transient spatial–temporal dynamics of gene expression and subtle differences across the genome are usually masked by cellular heterogeneity. With single-cell multi-omics approaches, individual cells are sampled and compared among populations, providing much more detailed information for an in-depth dissection of the precise intercellular regulatory mechanisms. In this section, we discuss the current application areas of single-cell-based studies. A schematic diagram is provided to better demonstrate the application areas of single-cell multi-omics approaches (Fig. 2).

### Plant tissue/organ atlas

Single-cell approaches are most commonly used to construct a cell composition map (atlas) of plant tissues or organs, aiming to understand the different plant cell types with respect to location-to-function information (Rhee et al. 2019). Taking advantage of single-cell isolation techniques and the known cell-type-specific marker genes, the cell atlas of *Arabidopsis* root has been constructed by several research groups (Denyer et al. 2019; Jean-Baptiste et al. 2019; Shahan et al. 2022; Shulse et al. 2019; Turco et al. 2019; Zhang et al. 2019). These studies applied various scRNA-seq methods to generate a root cell map and managed to identify underrepresented cell types, i.e., protophloem sieve elements (PSEs), lateral companion cells (CCs), phloem pole pericycle (PPP) cells, and metapholem sieve element (MSE) cells. Further analysis enabled the identification of novel marker genes and key regulators such as *DNA-BINDING WITH ONE FINGER* (*DOF)* and *PINEAPPLE (PAPL)* (Otero et al. 2022). A recent study generated a more comprehensive *Arabidopsis* root cell atlas consisting of more than 96,000 cells and 90% of all protein-coding genes (Shahan et al. 2022). In addition, the single-cell transcriptome atlas of *Arabidopsis* leaf vasculature was generated, revealing fundamental differences in amino acid metabolism activity and transport pathways between phloem parenchyma (PP) and CCs (Kim et al. 2021). Transcriptomic atlas of roots was also reported in rice and maize, which led to the discovery of the unique localization and mobility features of maize SHORT-ROOT (SHR) in the cortex cells (Ortiz-Ramírez et al. 2021), fungal response mechanisms in maize root tips (Cao et al. 2023), and cell-type-specific regulatory programs, including phytohormone biosynthesis, signaling, and response, in rice (Liu et al. 2021b; Wang et al. 2021). Characterization of the single-cell transcriptome of maize shoot apical meristem (SAM) led to the identification of the stem cell cluster, and revealed that genes involved in DNA damage repair, methylation, and genome stability maintenance are significantly upregulated in stem cells, providing insights into stem cell function and fate acquisition (Satterlee et al. 2020). A high-resolution single-cell transcriptome atlas of maize ear inflorescences was generated, and regulatory networks and candidate genes that control traits for ear yield were identified (Xu et al. 2021).

### Developmental trajectory reconstruction

Another common application area of single-cell sequencing approaches is tracking the differentiation and developmental trajectories of cell lineages. By combining the scRNA-seq and live-cell imaging data, the developmental patterns of *Arabidopsis* root phloem vasculature was precisely dissected from cell birth to enucleation. The bifurcation of the protophloem to form a metapholem sieve element (MSE) and procambium revealed the importance of *RHO in PLANTS* (*ROP*) *GTPase* signaling. The *ROP GTPases* were found to be regulated by the linage-specific transcription factor PHOLEM EARLY DNA-BINDING WITH ONE FIUNGER (PEAR), which also promotes the transcription of *ALTERED PHOLEM DEVELOPEMNT* (*APL*) (Roszak et al. 2021). Another report used scRNA-seq to determine the ontogeny of *Arabidopsis* lateral root primordium cells, identifying seven populations of cells, formed during the early stages of lateral root organogenesis, and the *C-REPEAT BINDING FACTOR 3* (*CBF3*) gene, which was identified as a regulator of lateral root primordium ontogeny (Serrano-Ron et al. 2021). To precisely describe the initiation and early developmental trajectory of lateral root primordium cells, scRNA-seq was performed on gravity-induced root segments, which are enriched in lateral root primordium cells. A cluster of previously unknown cells, which were differentiated from the middle column sheath, were identified at the early stage of lateral root initiation. Regulatory genes expressed specifically in these cells were identified and functionally characterized (Gala et al. 2021).

Apart from root cell development, the developmental trajectory of stomatal guard cells was also elucidated by scRNA-seq analysis of FACS-enriched epidermal cells and stomatal lineage. The novel expression patterns of cell cycle regulators and the broadened functional roles of *SPEECHLESS (SPCH)* in the later stages of guard cell development were identified (Lopez-Anido et al. 2021). Furthermore, researchers conducted a cell census of rice pistils before fertilization through the use of snRNA-seq, and revealed cell heterogeneity between ovule- and carpel-originated cells (Li et al. 2023a). The floral meristem differentiation process was studied by combining snRNA-seq with microcopy-based 3D spatial reconstruction. Gene expression differences among meristematic domains were determined and confirmed by confocal imaging (Neumann et al. 2022). Furthermore, the developmental trajectories of *Arabidopsis* female germline (Hou et al. 2021), rice floret and inflorescence meristems (Zong et al. 2022), cotton (*Gossypium hirsutum*) fiber (Qin et al. 2022), and bryophyte gemmae and thalli (Wang et al. 2023b) were reconstructed using scRNA-seq. These studies revealed the gene regulatory networks during tissue/organ differentiation and shed light on further deciphering the mechanism of plant development. In addition, the spatial transcriptome technology scStereo-seq was recently used to dissect minute, but significant, differences in gene expression patterns between the lower and upper epidermal cells of *Arabidopsis*, which helped to uncover specific spatial developmental trajectories in vascular cells and guard cells (Xia et al. 2022).

### Phenotypic characterization

Dissecting cellular heterogeneity can enable the molecular characterization of mutant cell phenotypes Comparative single-cell transcriptomic analysis has been conducted on the root protoplasts of wild-type plants and *root-hair defective* (*rhd6*) and *glabra2* (*gl2*) mutants, which lack hair cells and non-hair cells, respectively. The undifferentiated precursor cells were identified and cell fate related genes were analyzed in mutant epidermal cells, leading to the discovery that *rhd6* and *gl2* mutants are unable to fully convert one epidermal cell type into another (Ryu et al. 2019). In another study, the phenotype of *shortroot* (*shr*) and *scarecrow* (*scr*) mutants was characterized by scRNA-seq. The mixed cell identity phenotype was reflected as both mutants also showed a reduction in the number of endodermal cells, protoxylem cells, and xylem pole and phloem pole pericycle cells. Further analysis of cell identity markers revealed the cortex-to-endodermis fate transition phenotype in the *scr* mutant, suggesting that *SCR* is required for endodermal identity acquisition (Shahan et al. 2022).

### Intercellular signaling pathway

Intercellular signaling is essential for controlling the differentiation of plant cells and their interaction with environmental stimuli and pathogens. Non-autonomous signaling pathways have long been known to play a role in phytohormone response and developmental regulation. A single-cell 3D transcriptomic map of brassinosteroid signaling in wild-type *Arabidopsis* root and brassinosteriod-insensitive mutants showed that brassinosteroid signaling does not affect cell volume and proliferation but regulates the orientation of cell division planes and anisotropic cell expansion through arabinogalactan-encoding genes via non-cell-autonomous mechanisms (Graeff et al. 2021). Furthermore, the intercellular mechanisms of cytokinin biosynthesis and signaling were also dissected in single-cell transcriptomics studies. By developing a single-cell expression atlas of *Arabidopsis* root, the target genes of the vascular TARGET OF MONOPTEROS 5/LONESOME HIGHWAY (TMO5/LHW) transcription factor complex were detected in the outermost root hair cells, suggesting an intercellular signaling pathway that regulates root hair development. Further exploration revealed that TMO5/LHW-dependent vascular cytokinin biosynthesis is activated under phosphate-limiting conditions, which promotes root hair fate transition via the transport of cytokinin and activation of TMO5/LHW target genes in epidermal cells (Wendrich et al. 2020). Follow-up studies profiled the transcriptional dynamics at single-cell resolution upon cytokinin treatment, revealing that the TMO5/LHW complex-based increase in cytokinin levels occurs via the cooperative activity of BGLU44 and LOG4 in xylem cells, which is balanced by the sequential activation of SHR and CYTOKININ OXICDASE3 (CKX3) in procambium cells (Yang et al. 2021). Time-lapse RNA-seq and scRNA-seq data revealed the rapid activation of jasmonate, ethylene, and reactive oxygen species (ROS) pathways in response to wounding, and showed that key factors, including *BLADE-ON-PETIOLE1/2* (*BOP1/2*), *PLETHORA3/5/7* (*PLT3/5/7*), and *ETHYLENE RESPONSE FACTOR115* (*ERF15*), are involved in de novo root regeneration (DNRR) from detached *Arabidopsis* leaves (Liu et al. 2022). Recently, researchers using scRNA-seq found that *WUSCHEL-RELATED HOMEOBOX 13* (*WOX13*) negatively regulates SAM formation from callus in *Arabidopsis*, thus uncovering the molecular mechanisms underlying fate specification (Ogura et al. 2023). The signaling pathways that plants use to adapt to environmental changes and pathogen infection have also been identified at a high resolution. Six immune regulatory networks controlling *Fusarium verticillioides* (*Fv*) pathogenesis in the major cell types of maize root tips at single-cell resolution were constructed (Cao et al. 2023). Exposing *Arabidopsis* to *Pseudomonas syringae* and profiling > 11,000 individual cells using scRNA-seq led to the identification of distinct pathogen-responsive cell clusters exhibiting transcriptional responses ranging from immunity to susceptibility, which resolved the cellular heterogeneity within an infected leaf (Zhu et al. 2023b). Moreover, the transcriptomes of *Hevea* leaves were characterized during early powdery mildew infection using scRNA-seq, revealing that the *HbCNL2* gene enhances the defense of rubber leaves against powdery mildew (Liang et al. 2023).

### Epigenetic regulation

The lifelong reprogramming of epigenetic modifications (i.e., DNA methylation, histone modifications, etc.) has been extensively studied in animals. With the improvement of single-cell epigenomic techniques, the dynamic changes in DNA methylation and histone modification patterns have been studied in plants. Using whole-genome BS-seq, higher CG methylation and lower CHH methylation were reported in germline cells as well as the shoot apical stem cells that entered the reproductive phase in *Arabidopsis* (Gutzat et al. 2020; Walker et al. 2018). Studies in rice demonstrated the RNA-dependent DNA methylation pathway mediated CHH hypermethylation in the reproductive SAMs, which account for the highly methylated transposable element (TE) regions in gametes (Higo et al. 2020). In addition, sc-BRIF-seq was performed on single maize microspores, which discovered that DNA methylation is similar among the four microspores within a single tetrad but differs significantly among tetrads, suggesting non-simultaneous DNA methylation reprogramming (Li et al. 2019).

The histone modification signature of *Arabidopsis* male gametophytes was studied by performing chromatin immunoprecipitation sequencing (ChIP-seq) on bulked germ cells. Widespread chromatin bivalency at the pre-existing regions of H3K4me3 and H3K27me3 modifications were reported in mature sperm cells (Zhu et al. 2023a). Global reprogramming of H3K27me3 marks in *Arabidopsis* sperm cells mediated by the H3.10 histone variant replacement and histone demethylation were also reported (Borg et al. 2020). The development of ChIP-seq assay at the single-cell level, like snCut&Tag, which has successfully been used to determine the H3K4me3 modification in 3,679 single nuclei isolated from rice seedlings (Ouyang et al. 2022), will accelerate the elucidation of histone modification reprogramming that occurs during plant development.

Gene function is significantly affected by chromatin accessibility and structure. Both scATAC-seq and sci-ATAC-seq have enabled the identification of chromatin patterns in different cell types, and open chromatin patterns in-turn reveal important regulatory sequences and associated transcription factors. Single-cell chromatin accessibility in *Arabidopsis* root was recently reported, and more than 8,000 regulatory elements were identified (Dorrity et al. 2021; Farmer et al. 2021; Tu et al. 2022). In maize, cis-regulatory atlas constructed by scATAC-seq showed that cell-type specific *cis*-regulatory elements (CREs) are enriched with enhancer activity and are located within the un-methylated long terminal repeat (LTR) retrotransposons. These maize cell-type specific CREs are regions for phenotype-associated genes, which are ideal targets for maize breeding programs (Marand et al. 2021). The scATAC-seq analysis of rice radicle tissue revealed differences in chromatin accessibility between the meristematic and elongation zones, suggesting chromatin-level reprogramming in root meristems during cell differentiation. Further characterization of transcription factor binding motifs revealed the enrichment of the binding motifs for bZIP*,* bHLH and GATA in the meristematic zone and that of MYB binding sites in the elongation zone (Zhang et al. 2021). In another study, chromatin accessibility in rice roots under normal and heat stress conditions was determined at the single-cell resolution, which revealed the cell-type-specific dynamic changes in chromatin accessibility in response to heat stress and led to the identification of heat shock-specific accessible chromatin regions (ACRs) in these cell types (Feng et al. 2022).

## Single-cell multi-omics studies in horticulture plants

Agronomic traits usually begin forming from the early developmental stages and are determined in specific cell types within a short time frame. High-resolution spatial–temporal dissection of gene regulatory mechanisms is essential for yield and quality improvement, which can be utilized in breeding. In this section, the applications and future directions of single-cell multi-omics studies in horticulture plants are discussed.

### Applications in fruit and vegetable crops

Isolation of individual SAMs and transcriptomic analysis at fine temporal resolution revealed novel short-lived gene expression programs that are activated before flowering, which determine the timing of SAM transition from the vegetative to the reproductive stage (Meir et al. 2021). Another study applied RNA-seq on tomato fruit subjected to LMD or hand dissection and generated a spatiotemporal transcriptome map, which uncovered the spatial progression of tomato fruit ripening along the longitudinal axis. Tissue-dependent regulation of DNA demethylation at promoters of ripening related genes was also observed to study spatial gene expression pattern (Shinozaki et al. 2018). Proteomic analysis of isolated cell types was conducted on tomato root and pericarp tissues, and confirmed the presence of different proteins in individual cell types undergoing normal development or exposed to environmental fluctuations (Liang et al. 2018; Potts et al. 2022; Zhu et al. 2016). In a recent study, the initiation process of shoot-borne roots was studied in tomato by mcSCRB-seq, which revealed that the shoot-borne roots originate from a small population of primary phloem-associated cells and identified LATERAL ORGAN BOUNDARIES DOMAIN (LBD) transcription factor, named SHOOT-BORNE ROOTLESS (SBRL), as the key regulator of shoot-borne root initiation (Omary et al. 2022).

In *Brassica rapa*, functional differences between palisade mesophyll cells (PMCs) and spongy mesophyll cells (SMCs) were detected by scRNA-seq, and many cell-type-specific marker genes were identified, expanding the knowledge of PMC and SMC differentiation during leaf adaxial-abaxial surface patterning and during leaf development and morphogenesis in leafy vegetables (Guo et al. 2022). In addition, the single-cell transcriptional landscapes of *B. rapa* leaf cells in response to heat stress conditions were generated by performing scRNA-seq, revealing the cellular heterogeneity of gene expression in response to high temperature. Homologous genes belonging to the three subgenomes of *B. rapa* exhibited different expression patterns in different cell types, which provided new insights into subgenome dominance effects at the cellular level (Sun et al. 2022).

Recently, the first single-cell atlas of strawberry (*Fragaria* × *ananassa*) was constructed using scRNA-seq, and the researchers characterized the distinct gene expression profiles of hydathode, epidermal, and mesophyll cells during the incubation period of *Botrytis cinerea* and revealed signals of the transition from normal functioning to defense response in epidermal and mesophyll cells upon *B. cinerea* infection (Bai et al. 2022). Additionally, researchers established the first snRNA-seq system for litchi (*Litchi chinensis*) apical bud at different developmental stages, completed the construction of the first litchi apical bud cell atlas, and identified the key cell populations for bud-flowering decision. The results of snRNA-seq, combined with RT-PCR, RNA in situ hybridization, and dot-blot hybridization, demonstrated that *LcFT1* and *LcTFL1-2* mRNAs, which regulate flower formation in litchi, can be transported from leaves to the SAM (Yang et al. 2023). Furthermore, by integrating snRNA-seq and spatial transcriptomics, the cell-type-specific gene expression dynamics during nodule development in soybean (*Glycine max*) was resolved at the single-cell level for the first time, and a set of rare cell subtypes involved in nodule maturation and nitrogen fixation were successfully identified in infected cells (Liu et al. 2023).

Metabolic analysis of single cells helps to better understand cell differentiation, aging, changes due to disease states, and response to xenobiotics and physical stimuli. Using LAESI-MS, analysis of single epidermal cells sampled from the onion bulb revealed age-dependent metabolic differences, providing insight into cellular development and response (Shrestha and Vertes 2009). Samples taken from a single cell of *Vicia faba* leaves using nano-ESI tips under a microscope were directly introduced into a mass spectrometer by infusion and subjected to MS/MS analysis. While abscisic acid (ABA) and jasmonoyl isoleucine (JA-Ile) were detected in the single cells of water- and wound-stressed leaves, they were almost undetectable in non-stressed single cells, which demonstrated that stress-induced accumulation of ABA and JA-Ile could be monitored using living single cells (Shimizu et al. 2015).

### Applications in ornamental plants

In poplar (*Populus* spp.), scRNA-seq analysis led to establishment of the transcriptional landscape of major cell types found in stems and the xylem at single-cell resolution and identified novel cluster-specific marker genes, thus helping to uncover the basic principles of vascular cell specification and differentiation in trees (Chen et al. 2021; Li et al. 2021; Li et al. 2023c; Tung et al. 2023; Xie et al. 2022). Based on cross-species analyses of single-cell clusters and overlapping trajectories, researchers revealed the highly conserved ray, yet variable fusiform, lineages across angiosperms (Tung et al. 2023). Additionally, a developmental trajectory analysis was used to reconstruct the process of fiber cell differentiation in *Bombax ceiba* and root cell fate determination in Moso bamboo (*Phyllostachys edulis*) at single-cell resolution (Cheng et al. 2023; Ding et al. 2023; Qin et al. 2022). Comparative analysis of the scRNA-seq data of *B. ceiba* and cotton confirmed that the additional cell division process in *B. ceiba* is a novel species-specific mechanism of fiber cell development (Ding et al. 2023; Qin et al. 2022). Additionally, single-cell transcriptome analysis revealed the particularity of bamboo basal root development and the role of *PheWOX13* involved in root growth (Cheng et al. 2023).

In previous studies, metabolic profiles have been obtained at higher spatial resolutions in ornamental plants. Secondary metabolites such as hypericin, pseudohypericin, and biflavonoids localized to the dark glands on *Hypericum* leaves, placenta, stamens, styli and pollen were analyzed using LDI-TOF–MS and LDI-MSI (Hölscher et al. 2009). The constituents of the petal pigments of wishbone flower petals were analyzed on a single-cell basis by a combination of laser microsampling and nano-flow liquid chromatography-ESI MS (LC–ESI–MS) techniques (Kajiyama et al. 2006), and the metabolites in daffodil bulb epidermal cells were examined on a single-cell basis using LAESI-MS (Shrestha and Vertes 2009). Metabolites, including neutral carbohydrates and amino acids, were detected in single bulb cells of tulip by the joint application of a cell pressure probe and a UV-MALDI system, providing deeper insights into the events during growth or stress responses (Gholipour et al. 2012). Metabolites in the native environment of Wilde Malva leaf stalk single-mesophyll-cell and that of *Pelargonium zonale* living single leaf, stem, and petal cells were measured by nano-ESI–MS, identifying thousands of specific molecules (Lorenzo Tejedor et al. 2012; Tejedor et al. 2009). Additionally, in situ metabolomic analysis of the leaf and stem tissues of *C. roseus* was also performed at the cellular level, which revealed subtle, but significant, differences between the biosynthetic capacity of the stem and leaf and successfully elucidated single-cell-specific localization patterns of terpenoid indole alkaloids (TIAs) in the leaf tissue (Fujii et al. 2015; Yamamoto et al. 2019, 2016). The newly reported single-cell transcriptomics and multi-omics datasets were used to build the first high-resolution single-cell expression atlas of *C. roseus* leaves, and revealed sequential cell-type-specific partitioning of the leaf monoterpenoid indole alkaloids (MIAs) biosynthetic pathway (Li et al. 2023b; Sun et al. 2023)*.*

### Applications in other horticultural plants

Developmental trajectories of different cell types in tea (*Camellia sinensis*) leaves were reconstructed and a cell- and development-specific metabolic pathway of catechin ester was identified, based on the transcriptomic atlas of 16,977 single cells generated using scRNA-seq (Wang et al. 2022). In peanut (*Arachis hypogaea* L.), the transcriptomes of 6,815 single leaf cells were mapped and the transcription factor interactions in the primordium-driven development of mesophyll and epidermal cells were determined, which uncovered that the palisade cells differentiate into spongy cells and the epidermal cells originated earlier than the primordium (Liu et al. 2021a). These single-cell transcriptome studies provide new insights into the precise regulation of metabolism and development, which are crucial for the quality of horticulture plant products.

Alfalfa (*Medicago truncatula*), a model legume, is used extensively to study the process of nodulation. Investigation of the early responses of alfalfa roots to rhizobia inoculation using snRNA-seq revealed the strongest differential regulation of rhizobium infection among the pericycle, cortex, endodermis, and root hair cells at 48 h (Cervantes-Pérez et al. 2022). In addition, the differentiation trajectories and biofunctions of symbiotic and un-symbiotic fate cells in the root nodules of alfalfa plants were determined using scRNA-seq. This study found that symbiotic fate cells and the internal un-symbiotic cells were involved in symbiotic nitrogen fixation, while the peripheral un-symbiotic cells were involved in nitrogen assimilation via asparagine synthesis (Ye et al. 2022).

The corolla of wild tobacco (*Nicotiana attenuata*) emits a bouquet of scents, each one of which is composed mainly of benzylacetone (BA). By employing the scRNA-seq technique to analyze 3,756 single cells isolated from the *N. attenuate* corolla limbs and throat cups at three different time points and by performing single-cell MS to identify BA synthase (NaPKS2) and reductase (NaAER1) in the corolla, Kang et al. (2022) uncovered that BA is synthesized mainly in the corolla epidermal cells and confirmed that expression of *NaPKS2* and *NaAER1* controls the synthesis of BA at the sub‐organ level.

### Challenges and perspectives

Recent advances in developmental biology have been made possible by multi-omics studies at single-cell resolution. However, progress in plants has been slow, owing to the tremendous difficulty in isolating protoplasts from most plant tissues and/or purifying oversize protoplasts by flow cytometry. Furthermore, it is particularly difficult to isolate protoplasts from the tissues of perennial woody plants, because of their rigid secondary cell walls. snRNA-seq does not require the protoplast isolation step, snapshots the cell status and has broadened the capabilities of single-cell research (Kalish et al. 2020; Liang et al. 2019; Cervantes-Pérez et al. 2022; Yang et al. 2023; Liu et al. 2023;). Although snRNA-seq represents a promising alternative for surveying the transcriptome of individual cells in organs and tissues, the number of transcripts in the nucleus is relatively small. Therefore, it remains to be determined whether snRNA-seq can be developed and used as a stand-alone method to determine the cell type heterogeneity of complex plant tissues. Additionally, a general limitation of single-cell analyses of horticultural plants is organs that show a high degree of anatomical complexity at maturity, with a relatively small proportion of some such as stem cells, which generates considerable technical variation and leads to incomplete and/or biased views of the transcriptome, proteome, or metabolome of the cell. Usually, researchers are unable to annotate several cell clusters, because of the lack of cluster-specific genes. Applying spatial transcriptomics will aid us in mapping the location of these cell clusters in the future. Nevertheless, the resolution of spatial transcriptomics needs to be improved. Future advancements in spatial transcriptomics with high resolution and sequencing depth will facilitate the investigation of gene expression in a positional context in complex tissues. Additionally, some horticultural plants, for example, *Lilium brownie*, have large genomes and complex molecular compositions. Therefore, there is room for improvement of the overall performance of single-cell proteomics, metabolomics, and/or multi-omics applications, especially from the perspective of developing efficient microsampling methods for different platforms and improving measurement throughput.

Because single-cell characterization technologies comprise a powerful new suite of methods for studying biological heterogeneity and promise to deliver a much deeper understanding of how organisms function as a unified collection of cell types, more studies will need to be conducted on horticultural plants in the near future. These studies would focus on, for example, constructing the cell atlases and developmental trajectories of the roots of carrot (*Daucus carota*), radish (*Raphanus sativus*), and *Brassica* species; uncovering the detailed cell differentiation process and regulatory mechanisms of tuberization at single-cell resolution in potato (*Solanum tuberosum*) and sweetpotato (*Ipomoea batatas*); reconstructing the developmental process of tendrils of some *Vitaceae* fruits and *Cucurbitaceae* vegetables at high resolution and studying the regulatory mechanisms of leaf-derived and shoot-derived tendrils; elucidating the regulatory mechanisms of trichome formation and development in horticultural plants; identifying more epigenetic regulatory mechanisms of fruit and seed development in horticultural plants; and characterizing the cell type- and developmental stage-specific, multi-layer regulation of sexual cell fate transition in many horticultural plants, including cucumber (*Cucumis sativus*), melon (*Cucumis melo*), watermelon (*Citrullus lanatus*), and zucchini (*Cucurbita pepo*). Unanswered questions in horticulture research can be re-examined by multi-layer studies. Furthermore, since snRNA-seq does not have the limitations of protoplast preparation and can provide precise information on the regulation of gene expression, the application of such techniques increased rapidly in recent studies and more single-nucleus based studies are foreseen in horticulture research. Ultimately, with continued refinement and maturation, single-cell multi-omics will become a powerful and widely used tool for better understanding the developmental biology of horticultural plants.

## Data Availability

Not Applicable to this article as no datasets were generated or analysed during the current study.

## Abbreviations

1cell-DGE: Single cell-digital gene expression
BRIF-seq: Bisulfite-converted randomly integrated fragments sequencing
CEL-seq2: Cell expression by linear amplification and sequencing
CE-MS: Capillary electrophoresis mass spectrometry
CUT&Tag: Cleavage under targets and tagmentation
DESI: Desorption electrospray ionization
Dip-C: Diploid chromatin conformation capture
FLASH-seq: Fast length adjustment of short reads sequencing
LAESI: Laser ablation electrospray ionization
LC–ESI–MS: Liquid chromatography-electrospray ionization-mass spectrometry
LDI-MS: Laser desorption ionization mass spectrometry
LDI-ToF–MS: Laser desorption ionization time-of-flight mass spectrometry
LMD: Laser microdissection
mcSCRB-seq: Molecular crowding single-cell RNA barcoding and sequencing
MS: Mass spectrometry
MSI: Mass spectrometry imaging
MALDI-TOF MS: Matrix-assisted laser desorption/ionization time-of-flight mass spectrometry
nanoLC-MS/MS: Nano-liquid chromatography tandem mass spectrometry
nanoESI-MS: Nano-electrospray ionization mass spectrometry
PHYTOMap: Plant hybridization-based targeted observation of gene expression map
scRNA-seq: Single-cell RNA sequencing
scATAC-seq: Single-cell assay for transposase-accessible chromatin with high-throughput sequencing
sci-ATAC-seq: Single-cell combinatorial indexing assay for transposase-accessible chromatin with sequencing
scBS-seq: Single-cell bisulfite sequencing
scDNase-seq: Single-cell DNase I hypersensitive site sequencing
scRNA-seq: Single-cell RNA sequencing
scStereo-seq: Single-cell spatial enhanced resolution omics sequencing
sciMET: Single-cell combinatorial indexed assay for the assessment of DNA methylation
scHi-C: Single-cell high throughput chromatin capture techniques
sciHi-C: Single-cell combinational index Hi-C
SIMS: Secondary ion mass spectrometry
SMART-seq2: Switching mechanism at 5’ end of the RNA transcript sequencing
snCUT&Tag: Single-nuclues CUT&Tag
snRNA-seq: Single nucleus RNA sequencing
snmC-seq: Single-nucleus methylcytosine sequencing
UV-MALDI: Ultraviolet matrix-assisted laser desorption/ionization
